# Supplementary material for: Co-Application of 1-MCP and Laser Microporous Plastic Bag Packaging Maintains Postharvest Quality and Extends the Shelf-Life of Honey Peach Fruit
Source: Foods. 2022 Jun 14;11(12):1733. doi: 10.3390/foods11121733 (PMC9222991; doi:10.3390/foods11121733)
Supplement: Supplementary file 1 [file foods-11-01733-s001.zip › foods-1738159-supplementary.pdf]

## Supplementary Materials

**Table S1** Alpha diversity index of fungus in “Feicheng” peaches

**Table S2** Relative abundance of fungus in “Feicheng” peaches (%)

**Table S3** Alpha diversity index of bacteria in “Feicheng” peaches

**Table S4** Relative abundance of bacteria in “Feicheng” peaches (%)

**Table S1.** Alpha diversity index of fungus in “Feicheng” peaches

| Samples | OTU numbers | ACE | Chao1 | Shannon | Simpson | Coverage |
|---|---|---|---|---|---|---|
| 0 | 113 | 115.03 | 113.88 | 1.78 | 0.23 | 1.00 |
| 1 | 46 | 46.00 | 46.00 | 1.41 | 0.31 | 1.00 |
| 2 | 54 | 54.61 | 54.50 | 1.82 | 0.21 | 1.00 |

Note: 0, control at time 0; 1, control after 35 d; 2, combination treatment after storing for 35 d.

**Table S2.** Relative abundance of fungus in "Feicheng" peaches (%)

| Number | Fungus genus | 0 | 1 | 2 |
|---|---|---|---|---|
| 1 | *Alternaria* | 57.71 | 31.74 | 9.47 |
| 2 | *Stilbella* | 21.35 | 4.21 | 1.4 |
| 3 | *Trichothecium* | 6.94 | 0 | 0 |
| 4 | *Acremonium* | 5.18 | 0 | 0.04 |
| 5 | *Aureobasidium* | 3.12 | 27.09 | 81.39 |
| 6 | *Holtermanniella* | 1.74 | 0.53 | 2 |
| 7 | *Issatchenkia* | 0 | 10.39 | 0.15 |
| 8 | *Coprinellus* | 0.02 | 3.45 | 0 |
| 9 | *Cladosporium* | 0.23 | 3.38 | 0.11 |
| 10 | *Sodiomyces* | 0 | 2.22 | 0 |
| 11 | *Talaromyces* | 0 | 2.22 | 0 |
| 12 | *Trichoderma* | 0 | 1.94 | 0 |
| 13 | *Pyrenochaetopsis* | 0 | 1.46 | 0 |
| 14 | *Mrakia* | 0.46 | 1.18 | 0 |
| 15 | *Rhodotorula* | 0.15 | 1.16 | 0.89 |
| 16 | *Meyerozyma* | 0.01 | 0 | 1.53 |
| 17 | *Golubevia* | 0.12 | 0.05 | 1.07 |
| 18 | *Sporobolomyces* | 0.51 | 0 | 0.51 |
| 19 | *Stemphylium* | 0.24 | 0 | 0.04 |
| 20 | *Fusarium* | 0.24 | 0.05 | 0 |
| 21 | *Liberomyces* | 0.19 | 0 | 0 |
| 22 | *Colacogloea* | 0.19 | 0 | 0 |
| 23 | *Sphaerulina* | 0.14 | 0 | 0 |
| 24 | *Filobasidium* | 0.11 | 0 | 0.07 |
| 25 | *Cryptococcus* | 0.09 | 0 | 0.18 |
| 26 | *Strobilurus* | 0.09 | 0.88 | 0 |
| 27 | *Vishniacozyma* | 0.05 | 0 | 0.13 |
| 28 | *Nigrospora* | 0.04 | 0.83 | 0 |
| 29 | *Cladophialophora* | 0.03 | 0.74 | 0 |
| 30 | *Aspergillus* | 0.02 | 0.95 | 0.04 |
| 31 | *Gibellulopsis* | 0.02 | 0.72 | 0 |
| 32 | *Fusicolla* | 0.01 | 0.83 | 0 |
| 33 | *Verticillium* | 0.01 | 0.28 | 0 |
| 34 | *Wickerhamomyces* | 0 | 0.83 | 0 |
| 35 | *Malassezia* | 0 | 0.56 | 0 |
| 36 | *Cutaneotrichosporon* | 0 | 0.46 | 0.03 |
| 37 | *Microcera* | 0 | 0.14 | 0 |
| 38 | *Irpex* | 0 | 0 | 0.17 |
| 39 | *Ochroconis* | 0 | 0 | 0.12 |

| | | | | |
|---|---|---|---|---|
| 40 | *Bulleromyces* | 0 | 0 | 0.11 |
| 41 | *Other genus* | 0.67 | 0 | 0.43 |
| 42 | *Unclassified* | 0.32 | 1.71 | 0.12 |

Note: 0, control at time 0; 1, control after 35 d; 2, combination treatment after 35 d.

**Table S3.** Alpha diversity index of bacteria in "Feicheng" peaches

| Samples | OTU numbers | ACE | Chao1 | Shannon | Simpson | Coverage |
|---|---|---|---|---|---|---|
| 0 | 253 | 259.15 | 262.71 | 0.65 | 0.78 | 1.00 |
| 1 | 25 | 35.03 | 39.00 | 0.70 | 0.55 | 1.00 |
| 2 | 32 | 34.94 | 34.00 | 1.10 | 0.38 | 1.00 |

Note: 0, control at time 0; 1, control after 35 d; 2, combination treatment after storing for 35 d.

**Table S4.** Relative abundance of bacteria in "Feicheng" peaches (%)

| Number | Bacteria genus | 0 | 1 | 2 |
|---|---|---|---|---|
| 1 | *Streptophyta* | 88.85 | 66.05 | 49.83 |
| 2 | *Pantoea* | 0.02 | 0.07 | 15.12 |
| 3 | *Phenylobacterium* | 0.01 | 0.46 | 0.58 |
| 4 | *Burkholderia* | 0.01 | 0.20 | 0.39 |
| 5 | *Pseudomonas* | 0.01 | 0.00 | 0.10 |
| 6 | *Nitrosomonas* | 0.34 | 0.00 | 0.00 |
| 7 | *Geobacter* | 0.18 | 0.00 | 0.00 |
| 8 | *Kuenenia* | 0.17 | 0.00 | 0.00 |
| 9 | *Bacillus* | 0.16 | 0.00 | 0.00 |
| 10 | *Terrimicrobium* | 0.11 | 0.00 | 0.00 |
| 11 | *Rhodanobacter* | 0.11 | 0.00 | 0.00 |
| 12 | *Other genus* | 0.65 | 0.20 | 0.57 |
| 13 | *Unclassified* | 9.38 | 33.02 | 33.41 |

Note: 0, control at time 0; 1, control after 35 d; 2, combination treatment after storing for 35 d.

**Figure S1** Appearance changes of “Feicheng” peaches before and after 35 d of storage at 5 °C. A, polyethylene (PE, thickness: 20 μm) packaging at time 0; B, 1-MCP+LMF packaging at time 1; C, double corrugated boxes (DCB) packaging after 35 d; D, PE packaging after 35 d; E, 2.0 μL $L^{-1}$ 1-MCP+PE packaging after 35 d; F, 2.0 μL $L^{-1}$ 1-MCP+LMF packaging after 35 d.

**Figure S2** Weight lost (A) and rot rate (B) of “Feicheng” peaches during storage at 5 °C. PE, polyethylene packaging; DCB, double corrugated boxes packaging; 1-MCP+PE, 1-methylcyclopropene+ polyethylene packaging; 1-MCP+LMF, 1-methylcyclopropene+laser microporous film packaging. Vertical bars represent the standard errors of three replications.

**Figure S3** Soluble solids (A), TA (B), AsA (C) and soluble pectin (D) contents of “Feicheng” peaches during storage at 5 °C. PE, polyethylene packaging; DCB, double corrugated boxes packaging; 1-MCP+PE, 1-methylcyclopropene+ polyethylene packaging; 1-MCP+LMF, 1-methylcyclopropene+laser microporous film packaging. Vertical bars represent the standard errors of three replications.

**Figure S4** Respiration rate (A) and $O_2^{\cdot-}$ production rate (B) of “Feicheng” peaches during storage at 5 °C. PE, polyethylene packaging; DCB, double corrugated boxes packaging; 1-MCP+PE, 1-methylcyclopropene+ polyethylene packaging; 1-MCP+LMF, 1-methylcyclopropene+laser microporous film packaging. Vertical bars represent the standard errors of three replications.

**Figure S5** CAT (A), POD (B), lipase (C) and LOX (D) activities of “Feicheng”

peaches during storage at 5 °C. PE, polyethylene packaging; DCB, double corrugated boxes packaging; 1-MCP+PE, 1-methylcyclopropene+ polyethylene packaging; 1-MCP+LMF, 1-methylcyclopropene+laser microporous film packaging. Vertical bars represent the standard errors of three replications.

**Figure S6** PG (A), β-galactosidase (B) and Cx (C) activities of "Feicheng" peaches during storage at 5 °C. PE, polyethylene packaging; DCB, double corrugated boxes packaging; 1-MCP+PE, 1-methylcyclopropene+ polyethylene packaging; 1-MCP+LMF, 1-methylcyclopropene+laser microporous film packaging. Vertical bars represent the standard errors of three replications.

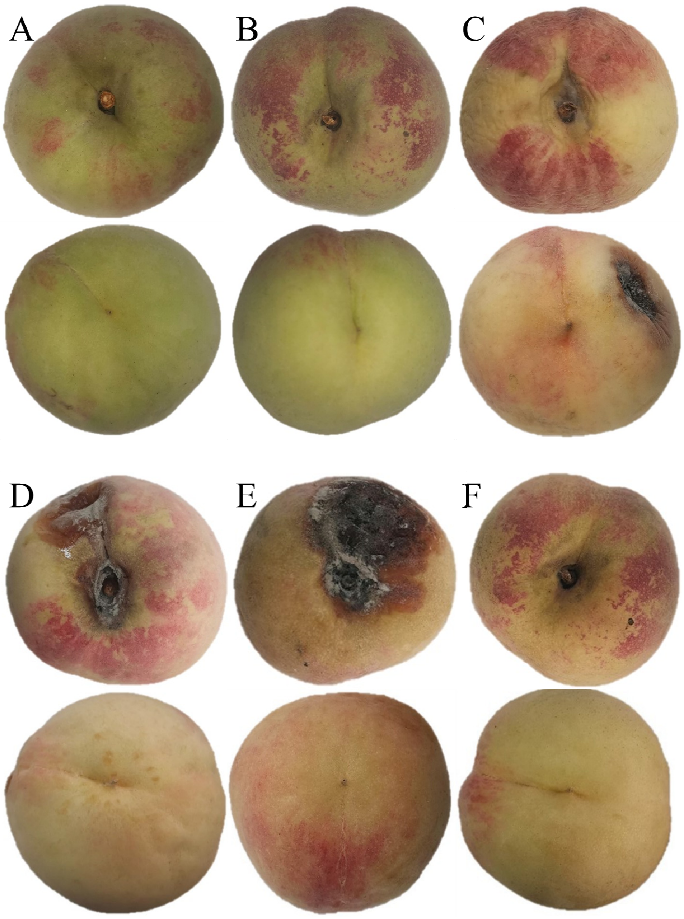


Figure S1. Appearance changes of “Feicheng” peaches before and after storing for 35 d at 5 °C. A, polyethylene (PE, thickness: 20 μm) packaging at time 0; B, 1-MCP+LMF packaging at time 1; C, double corrugated boxes (DCB) packaging after 35 d; D, PE packaging after 35 d; E, 2.0 μL $L^{-1}$ 1-MCP+PE packaging after 35 d; F, 2.0 μL $L^{-1}$ 1-MCP+LMF packaging after 35 d.

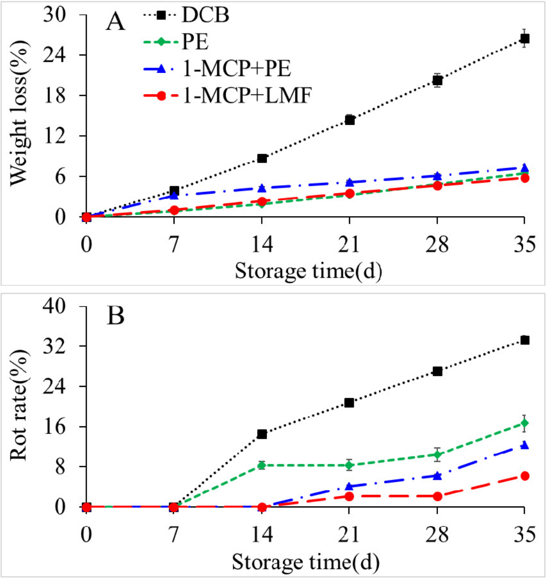


Figure S2. Weight lost (A) and rot rate (B) of "Feicheng" peaches during storage at 5 °C. PE, polyethylene packaging; DCB, double corrugated boxes packaging; 1-MCP+PE, 1-methylcyclopropene+ polyethylene packaging; 1-MCP+LMF, 1-methylcyclopropene+laser microporous film packaging. Vertical bars indicate the standard errors of three replications.

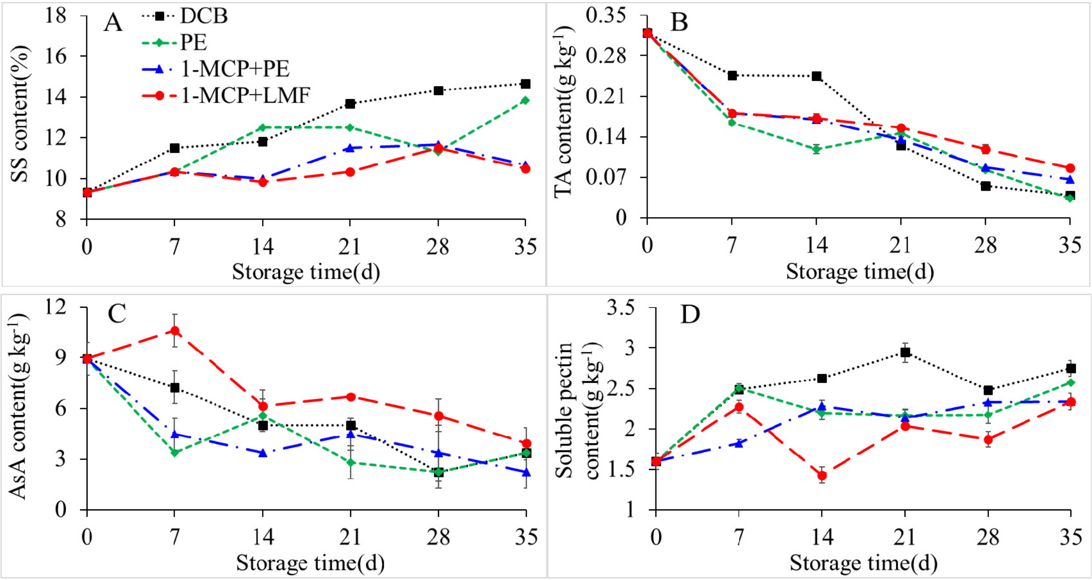


Figure S3. Soluble solids (A), TA (B), AsA (C) and soluble pectin (D) contents of "Feicheng" peaches during storage at 5 °C. PE, polyethylene packaging; DCB, double corrugated boxes packaging; 1-MCP+PE, 1-methylcyclopropene+ polyethylene packaging; 1-MCP+LMF, 1-methylcyclopropene+laser microporous film packaging. Vertical bars represent the standard errors of three replications.

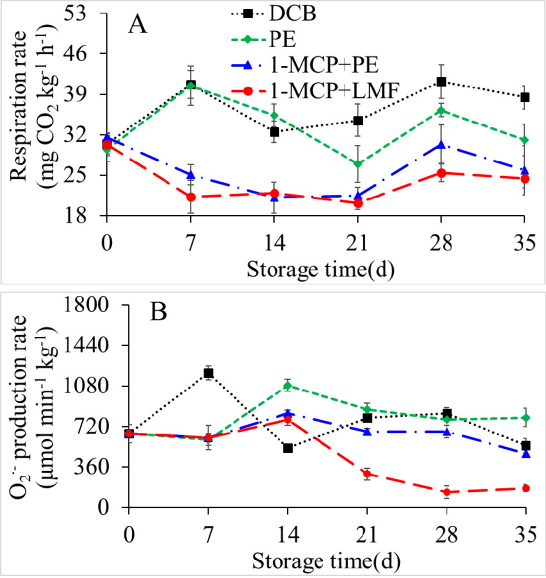


Figure S4. Respiration rate (A) and $O_2^{\cdot-}$ production rate (B) of “Feicheng” peaches during storage at 5 °C. PE, polyethylene packaging; DCB, double corrugated boxes packaging; 1-MCP+PE, 1-methylcyclopropene+ polyethylene packaging; 1-MCP+LMF, 1-methylcyclopropene+laser microporous film packaging. Vertical bars represent the standard errors of three replications.

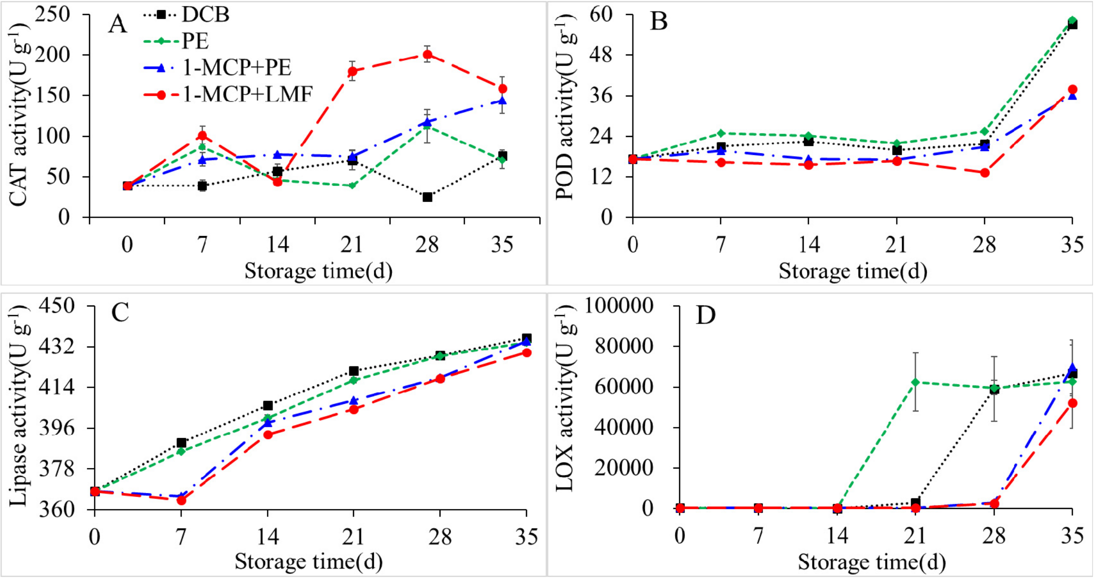


Figure S5. CAT (A), POD (B), lipase (C) and LOX (D) activities of "Feicheng" peaches during storage at 5 °C. PE, polyethylene packaging; DCB, double corrugated boxes packaging; 1-MCP+PE, 1-methylcyclopropene+ polyethylene packaging; 1-MCP+LMF, 1-methylcyclopropene+laser microporous film packaging. Vertical bars represent the standard errors of three replications.

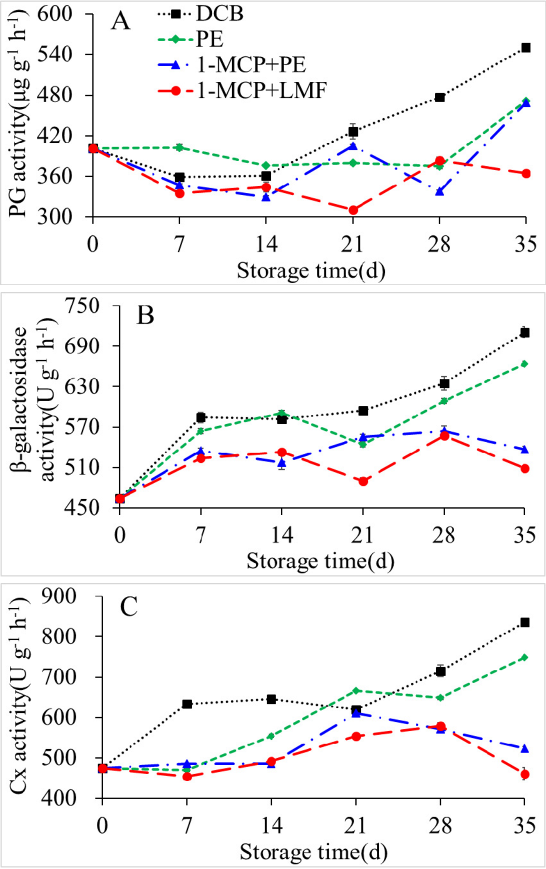


Figure S6. PG (A), β-galactosidase (B) and Cx (C) activities of "Feicheng" peaches during storage at 5 °C. PE, polyethylene packaging; DCB, double corrugated boxes packaging; 1-MCP+PE, 1-methylcyclopropene+ polyethylene packaging; 1-MCP+LMF, 1-methylcyclopropene+laser microporous film packaging. Vertical bars represent the standard errors of three replications.
